# Characterization of Changes in Gluten Proteins in Low-Gliadin Transgenic Wheat Lines in Response to Application of Different Nitrogen Regimes

**DOI:** 10.3389/fpls.2017.00257

**Published:** 2017-02-27

**Authors:** María Dolores García-Molina, Francisco Barro

**Affiliations:** Department of Plant Breeding, Institute for Sustainable Agriculture – Spanish National Research CouncilCórdoba, Spain

**Keywords:** transgenic wheat, promoters, gliadins, celiac disease, nitrogen, gluten, RP-HPLC, Competitive R5 ELISA

## Abstract

Gluten proteins are major determinants of the bread making quality of wheat but also of important gluten-related disorders. The gluten protein accumulation during grain filling is strongly influenced by nitrogen fertilization. We have characterized the gluten proteins in low-gliadin wheat lines as influenced by nitrogen treatments in two experiments. These transgenic lines, D783, D793, C655, D577, and E82 were obtained by using two different RNAi silencing fragments and two endosperm-specific promoters to drive the silencing fragments (d-hordein and γ-gliadin). In Experiment 1, we used three nitrogen fertilizer rates (120, 360, and 1080 mg N) added at sowing stage and combined with two sulfur rates (8 and 30 mg S); Experiment 2 included two nitrogen levels (120 and 1080 mg N), which were added according to the greatest demand per plant using split applications. The protein quantification was accomplished by Reverse-Phase High-Performance Liquid Chromatography and gluten content (ppm) determined using monoclonal antibody R5 (Competitive R5 ELISA). The results showed differences in protein accumulation between the two transgenic lines with the same silencing fragment but different promoter. Lines D793 and E82 showed low gliadin and an increment in glutenin content with increasing nitrogen. Competitive ELISA R5 showed a significant decrease in gluten content using split applications of nitrogen (Experiment 2) with 120 mg N compared to Experiment 1. In addition, line E82 ensures that variations in N fertilization will not result in increased gluten content.

## Introduction

Wheat is one of the most important crops in the world, providing energy and protein for humans and animals. Although starch is the major component of the grain, accounting for 60–75% of the total dry weight, proteins (9–15%) are very important for wheat functionality. Wheat flour is used to make a wide variety of products including cakes, pasta, bread, noodles, and biscuits, which is possibly because of the gluten proteins in wheat, which consist of gliadins and glutenins (Shewry et al., 2009). The gliadins, which provide extensibility and viscosity to the dough, are monomeric and they are classified into three structural fractions: α/b-, ω-, and γ-gliadins. The glutenins, responsible for dough elasticity, are present as polymeric complexes linked by disulphide bonds and comprise two types of subunits; the HMW and the LMW subunits of glutenin. Shewry et al. (1986) classified the wheat prolamins on the basis of their sulfur content into S-poor, consisting of ω-gliadins; S-rich composed of α- and γ-gliadins and the LMW-GS; and HMW-GS prolamins. Gluten proteins are also called prolamins because of their high content of the amino acids proline and glutamine (Shewry et al., 1995; Shewry and Halford, 2002). Gluten plays an important role in the final quality of wheat as its proteins form a cohesive viscoelastic network that allows the trapping of carbon dioxide during the fermentation and expansion of the dough, contributing to the final volume and texture of bread and other baked products.

Although wheat is a staple food in the human diet, gluten proteins are associated with three important pathologies and may affect up to 7% of the human population: (i) gluten allergy (Zuidmeer et al., 2008) (0.2–0.5% population), (ii) NCGS (Sapone et al., 2011) (6% population), and (iii) CD (Mariné et al., 2011) (1% population). CD is the one most studied and is an autoimmune disease in genetically predisposed individuals caused by the ingestion of gluten not only from wheat, but also from barley and rye (Trier, 1998). Moreover, 95 and 5% of CD patients who present, respectively, the genes encoding the human leukocyte antigen (HLA) DQ2 or DQ8 face a higher risk of developing this disease. Although the prevalence of CD is about 1% in Western countries (Farrell and Kelly, 2001), it is thought to be under-diagnosed. The only treatment available for CD patients is a lifelong strict GFD. However, it is difficult to follow a GFD as gluten is an important additive widely used in the food industry leading to transgressions in the diet that could affect between 32 and 55% of CD patients (Silvester and Rashid, 2007). Due to the presence of immunogenic proteins in rye (secalins) and barley (hordeins) (Comino et al., 2012), food products made with these cereals are also unsuitable for celiac-affected people. As a consequence of GFD, gut health may worsen and beneficial gut bacteria populations to be reduced (De Palma et al., 2009).

The use of genetic engineering, specifically RNAi technology, has been a promising approach to developing low-gliadin wheat varieties, and hence with reduced gluten-toxicity, which can be used as raw material for foods for gluten-intolerant people (Gil-Humanes et al., 2010, 2014a). These lines showed 90–98% reduction in the amount of gluten in comparison to the wild type, and they had reduced capacity for stimulating DQ2- and DQ8- restricted T-cell clones from celiac patients. The technological properties of doughs prepared using flour from the low-gliadin lines indicated a general weakening effect, although stability was increased significantly in some of the transgenic lines, suggesting better tolerance to over-mixing (Gil-Humanes et al., 2014b). It was possible to make breads using flour from low-gliadin lines, which showed baking and sensory properties, and overall acceptance, similar to those of normal flour, but with up to 97% lower gliadin content. Moreover, the low-gliadin flour has improved nutritional properties since its lysine content is significantly higher than that of normal flour (Gil-Humanes et al., 2014a).

Also, these lines can be useful for reducing the incidence of gluten-related pathologies as, in some cases, they have been associated with the amount and duration of gluten exposure (Ventura et al., 1999). Although the storage protein composition of these low-gliadin lines have been changed, reducing their toxicity, the total protein content is comparable to that of the wild type (Gil-Humanes et al., 2010, 2011; Pistón et al., 2011; Barro et al., 2016), suggesting a compensation mechanism with other proteins like albumins and globulins (Gil-Humanes et al., 2011).

The availability of sulfur (S) and nitrogen (N) in the soil through fertilization is a key factor for maintaining the protein profiles of wheat (López-Bellido et al., 1998; Wieser et al., 2004; Garrido-Lestache et al., 2005). S nutrition is essential to providing the cysteine residues that will form the disulphide bonds intra or inter-chain, playing an important role in gluten functionality (Shewry and Tatham, 1997). Several groups have showed a correlation between decreased S content in the soil and an increased proportion of ω-gliadins and HMW-GS linked to reduced α- and γ-gliadins, and LMW-GS (Wrigley et al., 1984; Fullington et al., 1987; Wieser et al., 2004) giving rise to a greater elasticity and lower extensibility of dough (Moss et al., 1983). Nitrogen is an essential plant nutrient required for high yield in wheat, and the source, amount of N and its application timings (Flæte et al., 2005; Fuertes-Mendizábal et al., 2010) are key factors directly correlated with yield. The nitrogen present in the grain determines the ratio of storage to non-storage proteins, the percentage of the storage protein fractions, and thus, the ratios of storage protein polymers to monomers. Therefore, adequate fertilization strategies by means of an optimal ratio between N and S are necessary to obtain a good combination of end-use protein quality, nitrogen use efficiency and high grain yield. Proteins make up about 80% of wheat plant organic S requiring one part of S per 17 parts of N, and plants try to maintain this ratio (Zhao et al., 1999a). Thus, N/S ratios greater than 17:1 will provoke S deficiencies induced by N and provide an accumulation of proteins non-rich in N such as amides, lower proportions of methionine and cysteine, and this leads to a decrease in wheat yield (Wrigley et al., 1980).

The availability of N and S during grain development influences the expression of prolamin genes (Giese and Hopp, 1984). Earlier studies have reported that the expression of prolamin genes is controlled at the transcription level, with a direct relationship between the number of transcripts and protein accumulated (Rahman et al., 1984; Bartels and Thompson, 1986). Transgenic wheat lines used in this study were obtained using a RNAi silencing fragment driven by two endosperm specific promoters; a d-hordein (Pistón et al., 2008) and a γ-gliadin (Pistón et al., 2009). Therefore, the expression of the RNAi silencing fragment, as well as target genes, could be greatly affected by fertilization, particularly N availability. Consequently, N fertilization of these lines is of great interest for the handling of the silencing efficiency by using the adequate balance of nutrients to obtain a reduction in gliadin toxicity, coupled with a good production.

The aim of the present work was to study how the controlled nitrogen conditions affect the silencing of gliadins and the accumulation of other storage proteins, specifically those triggering CD, and consequently, the toxicity in RNAi low-gliadin wheat lines. Results are important to minimize the accumulation of prolamins triggering CD but keep a good bread making quality.

## Materials and Methods

### Plant Material

Gil-Humanes et al. (2010) and Piston et al. (2013) have described previously the lines used in this work. A total of five transgenic lines from the *T. aestivum* cv. Bobwhite (denoted as Bobwhite 208), and the corresponding wild type were studied. These transgenic lines were obtained using different RNAi silencing fragments (ω/α- and γ-gliadin silencing fragments) and two endosperm-specific promoters to drive the silencing fragments (d-hordein and γ-gliadin promoters). **Table 1** summarizes the characteristics of these wheat lines.

**Table 1:** Characteristics of lines used in Experiments 1 and 2.

Genotype	Line	Constructs	Promoter	Prolamin target	Experiment
BW208	Wild type	NA	NA	NA	1, 2
	D783	pDhp_ω/α	D-hordein	α/β-, γ-, ω-	1, 2
	D793	pGhp_ω/α	γ-gliadin	α/β-, γ-, ω-	1, 2
	D577	pGhpg8.1	γ-gliadin	γ-	1
	C655	pDhpg8.1	D-hordein	γ-	1
	E82	pDhpg8.1+pDhp_ω/α	D-hordein	α/β-, γ-, ω-	2

*NA, non-applicable.*

### Fertilization Experiments

**FIGURE 1:**
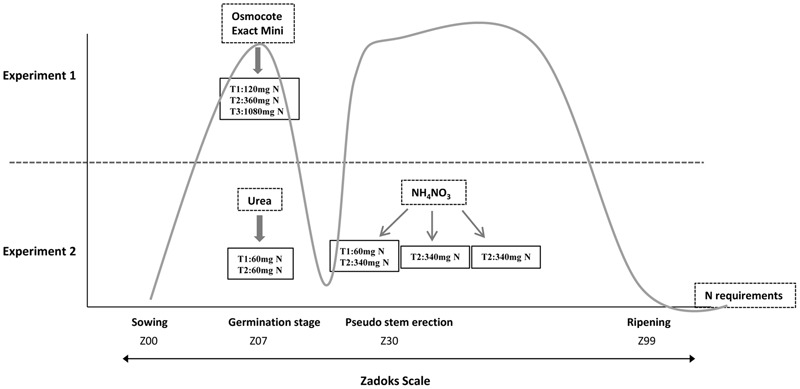
**Graphical representation of the different fertilization techniques and fertilization stages used in Experiments 1 and 2 based on N requirements.** T1, treatment 1; T2, treatment 2; T3, treatment 3; the gray line indicates nitrogen requirements throughout the development of the plant according to The Guidelines for Maximum Economic Yield-Wheat Production.

We carried out two fertilization experiments in a greenhouse under controlled temperature and humidity conditions (**Figure 1** and Supplementary Figure S1). To design these experiments, we considered a threshold fertilization level of about 200 kg N/ha and 20 kg S/ha., so amounts above and below those values were used. Both sets of experiments are described below.

#### Experiment 1

The first experiment consisted of three N fertilizer rates (120, 360, and 1080 mg per pot) in combination with two S rates (8 and 30 mg per pot) using a randomized complete block designs, with two plants per pot and line for each treatment and three blocks, comprising in all six plants per line and treatment. Each pot (1 L) was filled with arlite and vermiculite. Vermiculite is a mineral that has been superheated and expanded into thin layers. It has a good water-holding and exchange capacity, but, in order to reach an adequate growth, vermiculite must be mixed with arlite (lightweight expanded clay aggregate-LECA) due to it being necessary to maintain a reasonable pH favoring the drainage and oxygenation of the roots. Wheat grains were pre-germinated in a growing chamber at 4°C. When the coleoptile emerged from seeds, grains were sowed in this solid medium. Nitrogen and the rest of the nutrients, with the exception of sulfur, were applied at the beginning of the experiment using the commercial fertilizer ‘Osmocote Exact Mini’ (Scotts International B. V., The Netherlands), slow release fertilizer 16+8+11 (NPK) with magnesium and trace elements (**Figure 1**). The ‘Osmocote Exact Mini’ was added 2 days before planting and was mixed with the vermiculite to obtain a homogeneous substrate in the pots. To avoid additional input of N from irrigation water, we used distilled water. Sulfur was contributed by nutrient solution as calcium sulfate (CaSO_4_⋅2H_2_O). One week after sowing, wheat plants were irrigated with calcium sulfate solution twice a week (Supplementary Figure S1).

#### Experiment 2

The second trial was carried out with two N fertilizer rates organized as randomized blocks, with two plants per pot and line for each treatment and two blocks comprising in all four plants per line and treatment. Nitrogen fertilizer rates were 120 (Treatment 1) and 1080 (Treatment 2) mg N per pot. Accordingly, N treatments (Urea + Ammonium Nitrate) were 120 mg N (60 mg during sowing with Urea, +60 mg beginning the stem elongation with Ammonium Nitrate) and 1080 mg N (60 mg during sowing with Urea, +340 mg × 3 applications beginning the stem elongation with Ammonium Nitrate (one application per week)). These N fertilizer applications were carried out according to the greatest demand by the plant, that would correspond to the germination stage (growth stage 07) and pseudo stem erection (growth stage 30), according to the Zadoks scale (Zadoks et al., 1974) (**Figure 1**). The remaining nutrients were contributed through a nutritive solution. The concentration of nutrients in the final solution was that described by Spomer et al. (1997), i.e., about one-half the strength of Hoagland’s original solution (Hoagland and Arnon, 1950). To make this solution, we prepared a complete formulation of all macro and micronutrients using compounds without N in its composition and they were dissolved in distilled water. Supplementary Figure S1 shows the time and quantity of S fertilization during the wheat growth stages from Experiment 2, which was irrigated with this Hoagland solution once a week.

### Reversed-Phase High-Performance Liquid Chromatography (RP-HPLC)

Wheat samples were ground in a mixer mill (Star-Beater, VWR collection) to obtain flour of a 100 μm particle size. Gliadins and glutenins were extracted according to their solubility, using a modified Osborne procedure as described by Pistón et al. (2011). Briefly, gliadins were extracted from 100 mg of whole wheat flour with 670 μl of 60% (v/v) ethanol. After agitating the samples in a vortex shaker at room temperature for 10 min, the samples were centrifuged and the supernatant was collected. The process was repeated three times and the three supernatants were mixed all together. Glutenin fraction was extracted from the insoluble pellet using 500 μl of 50% (v/v) 1-propanol, 2 M urea, 0.05 M Tris-HCl (pH 7.5), and 2% (w/v) DTT. After vortexing and incubating for 15 min at 60°C, the supernatant was collected by centrifugation. The process was repeated three times. When the three supernatants were mixed, both extractions were filtered using nylon filter (0.45 μm). Two independent repetitions were made for each transgenic line and control. Quantitative determination was carried out using the RP-HPLC. Gliadin (60 μl) and glutenin (60 μl) extracts were applied to a 300SB-C8 reverse phase analytical column (4.6 mm × 250 mm, 5 μm particle size, 300 Å pore size; Agilent Technologies) using a 1200 Series Quaternary LC System liquid chromatograph (Agilent Technologies) with a DAD UV-V detector, as described in Wieser et al. (1998) and Gil-Humanes et al. (2010). In this chromatography, separation of the protein fractions is based on the hydrophobicity of each compound. Hence, the elution order will be ω-, α-, and γ- for gliadins, and HMW, LMW for glutenins (from lowest to highest hydrophobicity). The integration of the peaks was made automatically by the software. Bovine serum albumin (BSA; BSA ≥ 98%, fraction V. Sigma–Aldrich, St Louis, MO, USA, cat. no. A3294) was used as a standard protein to determine the absolute amounts of gliadins and glutenins. The areas under the curve of the UV-signal were calculated per μg of protein fraction/mg of flour to obtain the gliadin and glutenin contents for each sample.

### Competitive R5 ELISA

The analysis was carried out by Centro Nacional de Biotecnología using the R5 monoclonal antibody to detect gluten from transgenic and control lines, as described by Mena et al. (2012). The assay was performed in triplicate.

### Statistics

Data were analyzed using the Statistix software version 10.0 (Analytical Software, Tallahassee, FL, USA). Analysis of variance (ANOVA) with median multiple comparisons by LSD was used to analyze the results. *P-*values of less than 0.05 were considered to be significant.

## Results

### Experiment 1

Experiment 1 was used to evaluate the effects of three N treatments, with application of S at different stages as detailed in Supplementary Figure S1, on the gluten proteins of low-gliadin wheat lines (**Table 1**). Applications of S at different stages were analyzed and no significant differences were found. However, overall changes in gluten proteins, concerning all three N fertilization levels, both for μg protein/mg flour and for total grain protein (mg) accumulated per pot (**Table 2**) showed significant differences. Regarding protein content per flour unit (μg/mg flour), significant differences were found for ω-gliadins when increasing N from 120 to 360 mg, and from 120 to 1080 mg, but no differences were noted for α- and γ-gliadins (**Table 2**). Total gliadin content also increased significantly from 120 to 1080 mg N. With regard to the glutenins, only HMW and total glutenins showed significant differences from 120 to 1080 mg N. Overall, prolamin content (gliadins plus glutenins) increased significantly from 120 to 360 mg N and from 120 to 1080 mg N. Finally, the gliadin to glutenin ratio (Gli/Glu) was similar in all three N treatments.

**Table 2:** Storage protein content related to all three N treatments from Experiment 1.

	N treatment (mg)					
	**μg/mg flour**			**Total mg protein per pot**		
**Protein fraction**	**120**	**360**	**1080**	**120**	**360**	**1080**

ω-gliadins	13.5b	17.9a	18.6a	23.2b	60.2a	74.4a
α-gliadins	25.3a	32.3a	34.4a	44.2b	110.7a	139.8a
γ-gliadins	7.5a	9.1a	9.9a	14.5b	33.8a	39.9a
Total gliadins	46.3b	59.4ab	62.8a	81.8b	204.7a	254.0a
HMW	23.0b	25.9ab	27.9a	40.0c	79.8b	105.4a
LMW	25.4a	25.9a	28.8a	44.8c	82.5b	111.2a
Total glutenins	48.4b	51.9ab	56.7a	84.9c	162.3b	216.6a
Total prolamins	94.7b	111.3a	119.5a	166.7c	367.0b	470.6a
Gli/Glu ratio	0.96a	1.14a	1.11a			

*The values shown are referred to the average of the lines for each protein fraction. Gliadins and glutenins were determined by RP-HPLC. 120, 360, and 1080 are the N treatments expressed in mg of N. HMW, high molecular weight; LMW, low molecular weight.*

*Samples with same letter within each protein fraction are not significantly different at *p* < 0.05 by the LSD multiple comparison of means.*

The overall results, considering the total grain protein accumulated per pot (mg), also gave significant differences between the three treatments of N (**Table 2**). In this case, differences for all three gliadin fractions (ω-, α-, and γ-gliadins), and total gliadin content were significant when N was increased from 120 to 360 mg and from 120 to 1080 mg (**Table 2**). For HMW, LMW, total glutenin, and total prolamin, besides the above, were also significant when N was increased from 360 to 1080 mg.

The effect of the three levels of N was analyzed in detail for the wild type and all four transgenic lines (**Table 3**). As showed, for BW208 the N treatments were significant only for ω-gliadins, which increased from 16.3 to 20.4 μg/mg flour for 120 and 1080 mg N, respectively. In the case of D783 and D793, both containing a silencing fragment to target all gliadin fractions, the HMW and LMW, and also total glutenins and total prolamins were affected significantly when increasing N from 120 to 1080 mg N (**Table 3**). Gliadins were not affected, except ω-gliadins for line D793. In contrast for lines D577 and C655, both containing a silencing fragment to target only γ-gliadins, all gliadins fractions (except γ-gliadins for line C655) were significantly affected when N was increased from 120 to 360 and to 1080 mg. However, neither the glutenins fractions nor total glutenins were affected. Total gluten content (prolamins) was also increased significantly in lines D577 and C655with increasing N from 120 to 360 mg N and from 120 to 1080 mg N (**Table 3**). The gliadins to glutenins ratio increased also significantly from 120 to 360 and 1080 mg N for lines D577 and C655.

**Table 3:** Gliadin and glutenin contents (μg/mg flour), and gliadin to glutenin ratio of low gliadin transgenic and wild type lines for three different nitrogen treatments from Experiment 1.

		Gliadins				Glutenins				
Line	N (mg)	ω	α	γ	Total	HMW	LMW	Total	Prolamins	Gli/Glu
**BW208**	120	16.3b	38.5a	22.6a	77.3a	16.7a	21.6a	38.2a	115.6a	2.03a
	360	19.2ab	36.8a	26.4a	82.4a	16.7a	21.6a	38.3a	120.7a	2.21a
	1080	**20.4a**	38.9a	27.3a	86.6a	17.7a	22.6a	40.4a	127.0a	2.18a
**D783**	120	8.3a	16.1a	6.2a	30.6a	31.5b	33.3b	64.8b	95.5b	0.47a
	360	8.0a	16.1a	5.6a	29.8a	36.1ab	36.3ab	72.4ab	102.2ab	0.42a
	1080	9.0a	18.4a	6.1a	33.5a	**39.8a**	**42.2a**	**82.0a**	**115.5a**	0.41a
**D793**	120	7.4b	6.8a	0.6a	14.8a	22.9b	13.3a	36.2b	51.0b	0.42a
	360	7.9b	5.5a	1.0a	14.5a	**30.4a**	15.6a	46.1ab	60.5b	0.34a
	1080	**10.4a**	9.9a	2.4a	22.6a	**34.5a**	**18.3a**	**52.8a**	**75.5a**	0.43a
**D577**	120	18.2b	32.4b	3.9b	54.5b	22.5a	28.5a	51.0a	105.5b	1.08b
	360	**27.8a**	**52.0a**	6.0ab	**85.8a**	23.4a	26.2a	49.6a	**135.5a**	**1.81a**
	1080	**26.9a**	**53.5a**	**7.6a**	**88.1a**	23.9a	29.6a	53.5a	**141.6a**	**1.69a**
**C655**	120	17.5b	32.6b	4.2a	54.3b	21.2a	30.4a	51.6a	105.9b	1.06b
	360	**26.7a**	**51.3a**	6.5a	**84.5a**	23.0a	30.0a	53.0a	**137.5a**	**1.67a**
	1080	**26.1a**	**51.4a**	5.8a	**83.4a**	23.5a	31.1a	54.5a	**137.9a**	**1.55a**

*Gliadins and glutenins were determined by RP-HPLC. HMW, high molecular weight; LMW, low molecular weight.*

*Means with the same letter for each line and protein fraction are not significantly different as determined by LSD multiple comparisons at *p* < 0.05.*

The effect of the three levels of N on grain protein composition for transgenic and wild type lines considering the total grain protein per pot (Supplementary Table S1) was also evaluated. Hence, all gliadins fractions and total gliadins, in BW208 (wild type) and lines with silencing only of γ-gliadins (D577 and C655), increased significantly when N fertilization increased from 120 to 360 mg, and from 120 to 1080 mg N, but not for 360 to 1080 mg N. In contrast, for lines D783 and D793, this increment was only significant when N was increased from 120 to 1080 mg, except for γ-gliadins in the line D783, for which this increment was not significant (Supplementary Table S1). The glutenin fractions, total glutenins and total prolamins were also significantly affected on all lines when N was increased from 120 to 1080 mg. In addition, this increment was also significant from 120 to 360 mg N for lines D577 and C655 (Supplementary Table S1).

**FIGURE 2:**
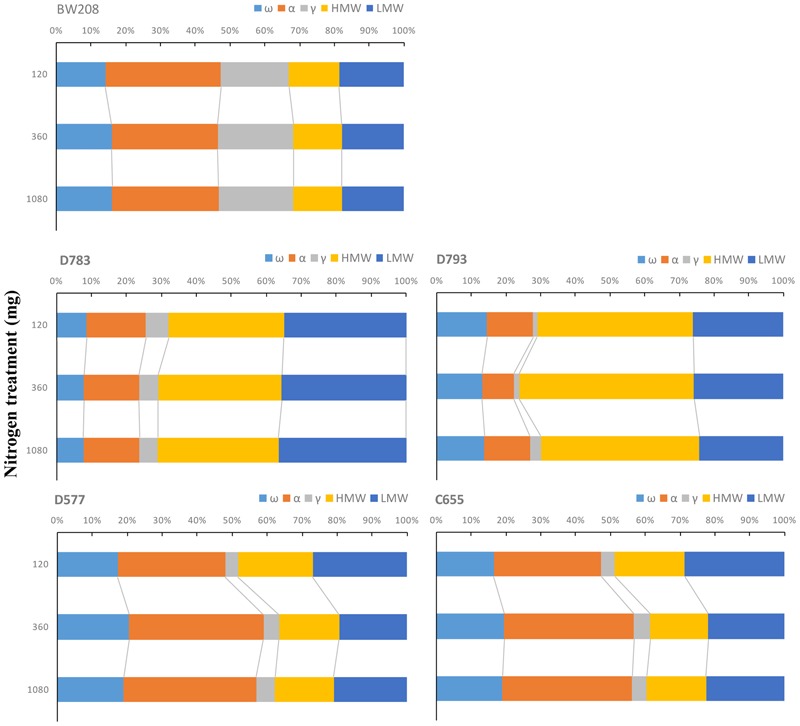
**Changes in the proportions of storage proteins in relation to total prolamin content as affected by three nitrogen treatments.** ω-, ω-gliadins; α, α-gliadins; γ-, γ-gliadins; HMW, high molecular weight; LMW, low molecular weight.

As consequence of N fertilization, the distribution of gluten protein changed among transgenic and wild type lines. Kernel distribution of gluten proteins is important for technological properties of doughs and it was studied in all lines. **Figure 2** shows how the proportions of the protein fractions change with N fertilization per flour unit. For BW208, the glutenin fraction represents about 35% of the total prolamin content. Increasing N from 120 to 360 mg increases the proportion of ω-gliadins and γ-gliadins but reduces α-gliadins (**Figure 2**). Both lines D783 and D793 showed similar behavior but with appreciable differences. With a lower N treatment rate (120 mg) the glutenin fraction represents about 70% of total storage proteins for line D783 (32% HMW and 35% LMW) and more than 70% for line D793 (45% HMW and 27% LMW). Increasing N from 120 to 360 mg increases the total glutenin fraction by increasing the HMW, and this increment is higher for line D793. The proportion of α-gliadins is strongly reduced in line D793 and less so in line D783. Increasing N from 360 to 1080 mg has no major effects on the protein distribution for line D783 but reduces the HMW and increases the α- and γ-gliadins proportions in line D793 (**Figure 2**). Lines D577 and C655 also showed comparable changes in the protein distribution. For these lines, the total glutenins represent about 50% using the lower N treatment rate (120 mg N). However, this proportion decreased to about 40% when N was increased from 120 to 360 mg and from 120 to 1080 mg by mainly increasing the proportions of α- and ω-gliadins (**Figure 2**).

**FIGURE 3:**
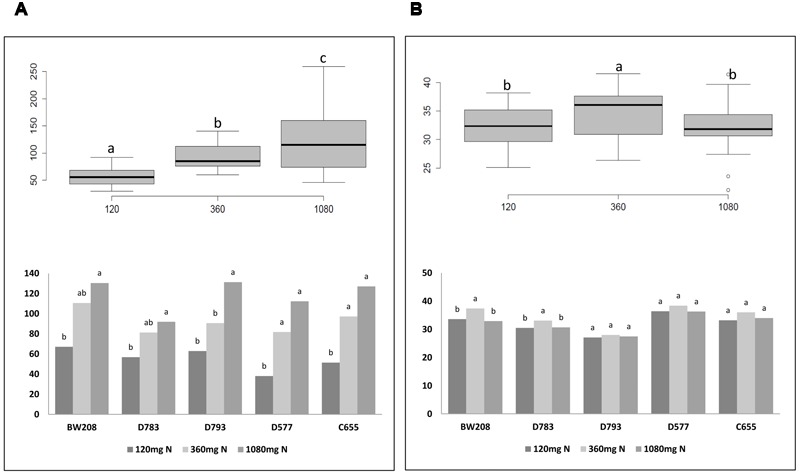
**Number of grains (A)** and kernel weight (g) **(B)** from Experiment 1. Box plots figures represent the number of grains per plant **(A)** and kernel weight **(B)** with respect to N fertilization; bar charts describe the number of grains per plant **(A)** and kernel weight **(B)** from each line with N fertilization. Means with the same letter for each line and N treatment are not significantly different as determined by LSD multiple comparisons at *p* < 0.05.

It is known that N is the main determinant of cereal grain quality and yield. The number of grains and kernel weight are depicted in **Figure 3**. Overall, the number of grains increased with N treatments, and the analysis of variance indicates significant differences between the three N treatments (**Figure 3A**, box plot). However, differences for kernel weight were only significant when N was increased from 120 to 360 mg (**Figure 3B**, box plot). Detailed analysis of all lines showed that the increment in the number of grains was significant for all them when N was increased from 120 to 1080 mg, and from 120 to 360 mg only for lines D577 and C655 (**Figure 3A**, bar chart). Regarding kernel weight, differences were significant when N was increased from 120 to 360 mg only for wild type and line D783 (**Figure 3B**, bar chart). For these, lines the higher values were obtained using 360 mg N.

**FIGURE 4:**
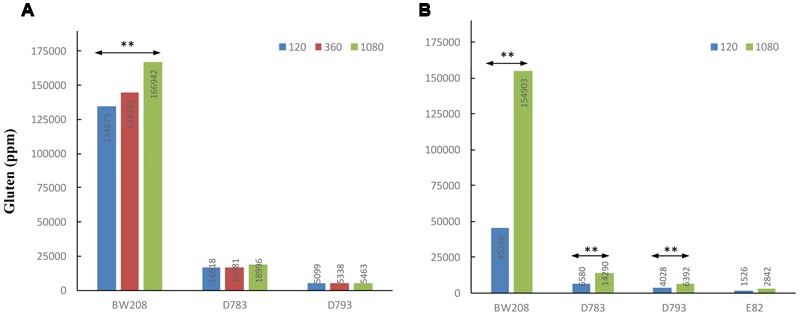
**Gluten content (ppm) by ELISA R5 of wild type and low-gliadin transgenic lines with different levels of nitrogen (mg N) from Experiments 1 (A)** and 2 **(B)**. ^∗∗^*p* < 0.01, means are significantly different as determined by LSD multiple comparisons of means.

To know how N affects the total gluten content (ppm), it was determined by the R5 monoclonal antibody and the results are showed in **Figure 4A**. R5 is the reference technique of Codex Alimentarius for quantifying the gluten content of foods. The determination of gluten content in ppm by R5 was carried out only in the wild type and transgenic lines D783 and D793, which contain the ω/α-gliadin silencing fragment as they had lesser contents of gluten (Gil-Humanes et al., 2010). Moreover, they are promising lines as estimations of maximum tolerable daily intake of bread, made using flour from these lines indicated that they could be consumed by people with CD (Gil-Humanes et al., 2014a). As expected, gluten content of low gliadin lines was lesser than that of the wild type whatever N treatment was used (**Figure 4A**). Gluten content increased for all three lines when N was increased. However, this increment was only significant for line BW208 (wild type) when N was increased from 120 to 1080 mg.

### Experiment 2

For this experiment 120 and 1080 mg N, as in Experiment 1, were used. In addition, we designed this experiment considering the N requirements of the plant during the development stage (**Figure 1**). Thus, we decided to provide the same basis of N in both treatments mixing the inert substrate with Urea that provides 46% of N; therefore, all plants would have the same levels of N during the tillering stage. The remaining N to reach the specified values was provided using Ammonium Nitrate (34.5% N) as indicated in **Figure 1**.

Overall changes in storage proteins, both for μg protein/mg flour and for total grain protein (mg) accumulated per pot are showed in Supplementary Table S2. Significant differences for all protein fractions and total prolamins were found between treatments at 120 and 1080 mg N. Detailed analysis for each line and treatment is in **Table 4**. The analysis of variance for gliadin and glutenin content, and total prolamins (μg/mg flour) for both wild type (BW208) and transgenic lines, indicated that wild type and line D783 presented highly significant differences for all protein fractions when increasing N from 120 to 1080 mg. For both lines, γ-gliadins were those most affected, with increments of 3.2 and 3.0 times for lines BW208 and D783, respectively. For line D793, all glutenin fractions, ω-gliadins, and total gliadins increased significantly with the 1080 mg N treatment, providing a significant increment in the total prolamin content (**Table 4**). The other line included in this experiment, E82, which combines two different RNAi constructs (**Table 1**), was only affected, when increasing the N, in the ω-gliadin, HMW and total glutenins. The gliadin to glutenin ratio was only significantly increased for line BW208.

**Table 4:** Gliadin and glutenin contents (μg/mg flour) of low-gliadin transgenics and wild type lines for two different nitrogen treatments from Experiment 2.

		RP-HPLC								
		Gliadins				Glutenins				
Line	N (mg)	ω	α	γ	Total	HMW	LMW	Total	Prolamins	Gli/Glu
**BW208**	120	7.7	14.9	8.9	31.5	7.3	13.1	20.4	51.9	1.56
	1080	**21.7^∗∗∗^**	**42.6^∗∗∗^**	**28.8^∗∗∗^**	**93.1^∗∗∗^**	**19.4^∗∗∗^**	**28.1^∗∗∗^**	**47.5^∗∗∗^**	**140.6^∗∗∗^**	**1.96^∗∗∗^**
**D783**	120	7.7	7.5	1.8	17.0	14.4	16.2	30.6	47.6	0.55
	1080	**11.1^∗∗∗^**	**15.6^∗∗∗^**	**5.4^∗∗∗^**	**32.1^∗∗∗^**	**32.3^∗∗∗^**	**33.6^∗∗∗^**	**65.9^∗∗∗^**	**98.0^∗∗∗^**	0.49
**D793**	120	9.1	6.7	0.8	16.6	23.1	15.9	39.1	55.6	0.43
	1080	**11.1^∗∗^**	8.3	1.3	**20.7^∗^**	**32.8^∗∗∗^**	**19.7^∗∗^**	**52.5^∗∗∗^**	**73.3^∗∗∗^**	0.39
**E82**	120	8.6	5.2	0.3	14.2	16.6	10.7	27.3	41.5	0.53
	1080	**9.7^∗^**	5.6	0.4	15.7	**20.0^∗^**	11.0	**31.1^∗∗^**	46.8	0.51

*Gliadins and glutenins were determined by RP-HPLC. HMW, high molecular weight; LMW, low molecular weight.*

*Means are significantly different between treatments for each line and protein fraction, as determined by LSD multiple comparisons: ^∗^*p* < 0.05; ^∗∗^*p* < 0.01; ^∗∗∗^*p* < 0.001.*

The proportions of the protein fractions and how they change with N fertilization in relation to total prolamin content per flour unit is outlined in Supplementary Figure S2. As showed, total glutenins represented about 40% of total prolamins for the wild type (BW208) in the lower N treatment rate (120 mg). For all three transgenic lines the proportion of glutenins in the low N treatment rate was higher than that of BW208, ranging from about 65 to 70% for lines E82 and D783, and D793, respectively. Both E82 and D793 lines had comparable proportions of LMW to that of the BW208. All three transgenic lines had higher proportions of HMW than the wild type at lower N treatment. Increasing N reduced the proportion of LMW and increased the γ-gliadins in the wild type, while for transgenic lines the proportions of HMW were increased and the ω-gliadin fractions significantly decreased for line D783.

Supplementary Figure S3 shows the effects of N fertilization in Experiment 2 on the number of grains (A) and kernel weight (B). Overall, the number of grains increased with the increment of N fertilization. However, differences between N treatments were not significant for the number of grains (Supplementary Figure S3A, box plot). All lines, except the wild type, showed an increase in the number of grains with N fertilization but with no significant differences between treatments (Supplementary Figure S3A, bar chart). Kernel weight decreased significantly with N fertilization (Supplementary Figure S3B, box plot). In spite of all the lines tending to reduce their kernel weight when increasing N, differences were only significant for line E82 (Supplementary Figure S3B, bar chart).

The results obtained by R5 for gluten content (ppm) indicate that there are highly significant differences (*p* < 0.01) between treatments for all lines in this Experiment 2 except for line E82, which was not affected by the increment in the N fertilization (**Figure 4B**). For lines D783 and D793, and BW208, gluten content increased with N, and the wild type achieved the highest increase in ppm of gluten from 45266 ppm with 120 mg N to 154903 ppm with 1080 mg N.

### Protein Changes Comparison between Experiments 1 and 2

**FIGURE 5:**
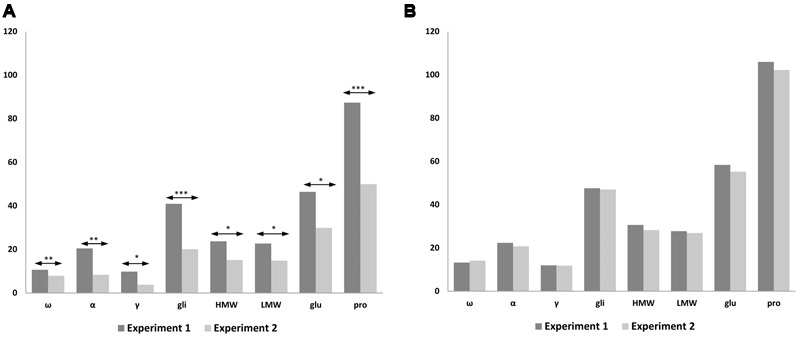
**Comparison of storage proteins from Experiments 1 and 2 using 120 mg N (A)** and 1080 mg N **(B)**. The bar chart shows the average values of the lines for each protein fraction; ω-, ω-gliadins; α, α-gliadins; γ-, γ-gliadins; gli, total gliadins; HMW, high molecular weight; LMW, low molecular weight; glu, total glutenins; pro, prolamins. Means are significantly different between experiments for 120 mg N; ^∗^*p* < 0.05; ^∗∗^*p* < 0.01; ^∗∗∗^*p* < 0.001; no significant differences for 1080 mg N.

We carried out a statistical analysis comparing N treatments of 120 and 1080 mg N from both Experiments 1 and 2, using the common lines present in both experiments, such as BW208, D783, and D793 (**Figure 5**). Significant differences were found between both experiments using 120 mg N (**Figure 5A**) but none when 1080 mg N was used (**Figure 5B**). As showed, significant differences were found for all protein fractions analyzed, which had higher values in Experiment 1 than in Experiment 2 with 120 mg N. Similar results were also found for gluten content as determined by R5 monoclonal antibody (**Figure 4**), which was much higher for the lower N treatment rate in Experiment 1 than in Experiment 2, but comparable results for 1080 mg N (**Figure 4**).

## Discussion

It is known that the type and amount of proteins accumulated during grain filling have a genetic component, but, also, the environmental growing conditions, particularly N fertilization, may cause changes in the quantity and distribution of these proteins. Wieser and Seilmeier (1998) reported that the composition of the protein fractions was more influenced by the N nutrition than by temperature. In this work, we analyzed the effect of N fertilization on the storage proteins of a set of wheat lines targeted by RNAi to down-regulate the gliadin fraction of gluten. Storage proteins, i.e., gluten proteins, are particularly important not only to the bread making quality of wheat but also to human health as they are responsible for serious food intolerances that may affect up to 7% of the population (Mariné et al., 2011; Sapone et al., 2011). Two experiments, with two N fertilization strategies, were designed to understand the response of these low-gliadin lines to N fertilization and the changes that occur in gluten proteins, particularly in the most CD immunogenic fractions and those related to bread making quality. We have evaluated the relationship between protein fractions for each line and N treatment; changes in grain yield, kernel weight, and grain production.

### Experiment 1

Globally, the three N fertilizations tested in this experiment had significant effects on the prolamin fractions (**Table 2**). This increase of the grain proteins based on the increment of N has previously been described (Wieser and Seilmeier, 1998; Garrido-Lestache et al., 2005; Xue et al., 2016). Moreover, in our case, these differences were also dependent on the genotype of the lines (**Table 3**). Thus, the transgenic lines D577 and C655 had a very similar behavior to those wheat lines described by Jia et al. (1996), in which there was an increase in the proportion of gliadins without changes in the glutenins when the N was increased. In the case BW208, only ω-gliadin fraction increased, according to Daniel and Triboi (2000), who obtained a slight increment in ω-gliadin fractions with increasing N. Similar results were found by Wieser and Seilmeier (1998) and they suggested that this increase in ω-gliadins might be due to the high level of glutamine that these fractions contain. However, in contrast to literature describing a decrease in α- and γ- gliadins (Daniel and Triboi, 2000), our results not showed significant differences in these gliadin fractions.

Differences in the down-regulation of gliadins of each line used in this study were depending on plasmid combination (**Table 3**), which confirmed results previously reported (Gil-Humanes et al., 2010; Piston et al., 2013). In Experiment 1, the transgenic lines with only γ-gliadins silenced (D577 and C655) had a low content of those gliadin fractions in all three N treatments, but not the α- and ω-gliadins, which increased with N. In fact, our results show that the strong silencing is more stable in the line C655 than in D577 despite the change in fertilization, since α-, ω-, and total gliadins are modified, but γ-gliadins remain constant in line C655 and the total content of gliadins was comparable to that of the wild type line (**Table 3**). Line D577 presented a significant increase in γ-gliadins from 120 to 1080 mg N, although data were lower than those of the control in spite of no significant changes occurring in the wild type. Lines D783 and D793 kept their strong silencing pattern, except for ω-gliadins of line D793, and were not affected by the increment in the N fertilization. Moreover, the increase in N led to incrementing the glutenin fractions of lines D783 and D793, which could be desirable as increased dough elasticity and toughness, thus reducing the energy required for mixing, have been described when increasing glutenins (Zhao et al., 1999b; Wooding et al., 2000a,b; Flæte et al., 2005). A possible explanation to clarify these results could be that changes in specific protein fractions produced by N fertilization are related to silencing targets used to generate the lines of this study (Gil-Humanes et al., 2010; Barro et al., 2016). Hence, in lines with the γ-gliadin target (D577 and C655), there was an increase in total gliadins without any change in glutenins. In the case of lines with α/β-, γ-, ω-gliadin target (D783 and D793) an increment in glutenins was obtained without increasing total gliadins. As a consequence, the gliadin to glutenin ratio has been increased by N fertilization in D577 and C655 without modifications in lines BW208, D783, and D793.

On the other hand, all gliadins and glutenins fractions accumulated in the grain were affected in response to N fertilization (Supplementary Table S1). In this case, all lines tended to increase the grain protein content from 120 to 1080 mg N. This may be because, in Experiment 1, the total quantity of N, for each treatment, was added at the beginning (**Figure 1**), resulting in a direct relationship between increased grain production, and increased N fertilization (*p* < 0.05) (**Figure 3A**). These findings would be in agreement with Van Herwaarden (1996) supporting that plants use available N to increase tillering and the remaining N is targeted to synthesize the wheat proteins. In this way, plants that had received less N used up less N in the grain production, and the amount of N used for protein synthesis in the grain was practically equal to that employed by the plants fertilized with high N rate (**Table 3**). In agreement with Oscarson (2000), who found a direct relationship between N and number of grains, this becomes clear when the grain production is compared between all three N treatments. Increasing N tends to increase grain production, with significant differences between all treatments. According to Oscarson (2000), in our study the grain weight was not modified with increasing N. It might indicate that the N availability gave rise to reservoirs directed to higher tiller production and more number of grains. Moreover, the kernel weight even tended to decrease from 360 to 1080 mg N, agreeing with what was described by Van Herwaarden (1996) (**Figure 3B**).

Regarding the gluten content (ppm) determined by monoclonal antibody R5, the results of Experiment 1 (**Figure 4A**) showed an increment in gluten content (ppm) as a result of N supplies for the wild type (BW208), but in the case of lines D783 and D793, no differences between N treatments and gluten content were found. Based on these results, and corroborating them with **Table 3**, the gluten (ppm) values for lines D783 and D793 are related to lack of changes in the gliadin fractions with increment of N. The strong silencing of gliadins in lines D783 and D793 prevents the accumulation of toxic epitopes by increasing N, since gliadins, mainly α-gliadins, are considered to be potentially toxic for patients with CD (Jabri and Sollid, 2006). **Figure 2** shows that, in lines D783 and D793, the α-gliadins fraction largely decreases when N is increased from 120 to 360 mg N, and this is coupled with an increase in HMW. The increment of glutenins, without increasing toxicity, is of a great significance for celiac food product preparation as they may/could conserve good nutritional and rheological properties with low-gliadin content.

### Experiment 2

This experiment was developed on the basis of results obtained during Experiment 1 using two of the lines D783 and D793, one new transgenic line (E82) and the wild type (BW208). Line E82 contains a combination of the two silencing fragments used in the experiment I. Therefore, in the light of results from Experiment I, this combination of fragments will provide essential information of how both fragments can work together. Storage protein content reached the largest differences in experiment 1 comparing 120 and 1080 mg N (**Table 2**). So, we decided to fertilize only with these amounts and split N applications as reported by Garrido-Lestache et al. (2004), who agreed with an improved wheat quality linked to splitting N. The fertilization design included the supply of ammonium nitrate at different stages to avoid any of its toxic effects on plants as described in Cox and Reisenauer (1973).

Overall, in this experiment there were differences, both per flour unit and total grain N per pot between treatments, indicating that even with different fertilization strategies, a higher N rate provides a greater accumulation of all gliadin and glutenin fractions and total prolamin content (Triboï et al., 2003; Matre et al., 2006) (Supplementary Table S2).

Lines used in this study exhibited different gliadin profiles as a consequence of the specific silencing by RNAi fragments and promoters. The storage protein modifications induced by fertilization were different, depending on the line. The reduction of gliadins, in lines D783 and D793, was reported as being 69.8 and 87.9%, respectively (Gil-Humanes et al., 2010), suggesting that line D793 had a greater potential of silencing that line D783. **Table 4** showed that the high N rate supplied in line D793 had a lesser effect on the gliadins composition than for D783. Only ω- and total gliadins increased with N in D793, while all glutenin fractions increased with increasing N in both D783 and D793 lines. Line E82, which was developed with a combination of two silencing fragments (γ-gliadin + ω/α-gliadin), displayed the highest level of down-regulation of gliadins (Gil-Humanes et al., 2010). This line showed the most unaltered protein fraction profile whatever N treatment was used, and only the ω-gliadins, HMW and total glutenins were amended with increasing N (**Table 4**). Furthermore, in agreement with Xue et al. (2016), our results confirmed that split N fertilizations affected the distribution of N in the grain, resulting in an increase of both gliadins and glutenins. This could be the result of the way in which the amino acids are exported to the phloem for the synthesis of the proteins. One of the two reservoirs destined to provide the amino acids to the phloem is fed through the N absorbed by the plant. The second reservoir, which would act as a store, accumulates the amino acids in stages in which the N is not limiting and, when the first reservoir is depleted, it releases those amino acids slowly (Barneix, 2007). Moreover, in our study both HMW-GS and LMW-GS increased with split N applications, in contrast to other studies (Daniel and Triboi, 2000; Shewry et al., 2001; Hurkman et al., 2013) who described an increment in the low S-proteins (HMW-GS) and a decrease in the high S-proteins (LMW-GS) with N fertilization, suggesting that split N application could promote the N availability compared to S.

The gliadin to glutenin ratio was only increased by N fertilization in the wild type, which agrees with Kindred et al. (2008), who calculated the proportion of gliadins and glutenins using SE-HPLC, and they found that increasing grain protein by N fertilization is associated with increased proportion of gliadins, and, thus, with an increase in the gliadin to glutenin ratio. Similar results have been reported by Wieser and Seilmeier (1998) and Daniel and Triboi (2000).

In relation to the gluten content (ppm) detected by R5 ELISA, the latter increased by increasing N rate for wild type (BW208), and lines D783 and D793 (**Figure 4B**). One explanation for this could be that, at 1080 mg N, the synthesis of grain proteins was favored instead of tillering and grain production, but not with 120 mg N, resulting in a direct relationship between N and gluten content (ppm). However, line E82’s gluten content was not affected (ppm) by N addition. This could be due to the great efficiency of the RNAi down-regulation in this line (Gil-Humanes et al., 2010; Barro et al., 2016). Supporting these results, Supplementary Figure S2 shows that lines D793 and E82 present similar proportions of the protein fractions in relation to total prolamin content. The difference between these two lines is that the total prolamin content is higher in line D793 than in E82 (**Table 4**). This increment in line D793 depends on ω- and total gliadins, and glutenins. Therefore, although the distribution of storage proteins is comparable between the two lines, the toxicity determined by R5 is different (**Figure 4B**). Regarding line D783 with 120 mg N, the prolamin content is intermediate between D793 and E82 (**Table 4**). This could indicate that the promoter of line D793 (γ-gliadin promoter) is more effective in the silencing of gliadins than that of D783 (d-hordein promoter). On the other hand, lines D783 and E82 share the d-hordein promoter, but the effectiveness of the silencing is higher in line E82, indicating that it is required to combine the two silencing fragments.

On the other hand, increasing N is not correlated with an increase in the number of grains or in the kernel weight (Supplementary Figure S3). This could also be due to the split N applications were used during the two periods of greatest need. Thus, each plant had the same N amount available during tillering (**Figure 1**), leading to no correlation between grain production and N. This fertilization strategy allowed the accumulation of different amounts of wheat proteins but not the increment in the number of grains and kernel weight.

In both experiments, N directed toward forming the prolamins of wheat was increased by increasing the amount of fertilizer, leading to higher gluten proteins. In agreement with Xue et al. (2016) describing that the increment of grain protein concentration is mainly influenced by the increase of N rather than split N applications, our results show that in both experiments the protein concentration using 1080 mg N is the same with and without split N applications (**Figure 5B**). However, the fertilization strategy could explain differences for storage proteins between Experiments 1 and 2, when 120 mg N was used (**Figure 5A**). During the wheat tillering, in Experiment 1 there was twice as much N as in Experiment 2 and therefore there was more N for protein synthesis. These results fit in well with gluten content (ppm) (**Figure 4**), and when using 1080 mg N (**Figure 4B**) gluten content for wheat lines (ppm) the latter are comparable between both experiments; however, when 120 mg N was used (**Figure 4A**), wheat lines had lower values of gluten content (ppm) in Experiment 2 than in Experiment 1, especially for lines BW208 and D783. In support of that, on comparing gliadin and glutenin contents from Experiment 1 (**Table 3**) and Experiment 2 (**Table 4**), it was observed that both BW208 and D783 accumulated about twice as much protein, considering all protein fractions, during Experiment 1 than in Experiment 2. In contrast, line D793 had comparable amounts of protein fractions in both experiments.

Moreover, previous studies found that the increment of metabolic proteins was not proportional to the N inputs, indicating that once the needs of the metabolic proteins are covered, the rest of the N is used to accumulate storage proteins (Johansson et al., 2001; Fuertes-Mendizábal et al., 2010). Our results fit this model well, since transgenic lines used in this study, presented increases in albumin and globulin fractions to compensate the suppression of gliadins and providing a similar total protein content to wild type (Gil-Humanes et al., 2011).

## Conclusion

Results indicated that providing 120 mg N, the gliadins, glutenins, and total prolamins accumulation during Experiment 1 was twice that of Experiment 2, and the gluten content (ppm) determined in Experiment 1 for BW208, D783, and D793 was 3.0, 2.5, and 1.3 times higher than that in Experiment 2, respectively. This could indicate that split N and by adding 120 mg N is desirable for a lower gluten content (ppm). Results obtained for the line E82 are of the great importance for N fertilization, as increasing N input will not result in increased gluten content (ppm), and therefore it would be easier to manage by farmers when the development of foodstuff for CD patients or other gluten intolerance group using these low-gliadin lines is intended.

## Author Contributions

Conceived and designed the experiments: FB and MG-M. Performed the experiments: MG-M. Analyzed the data: FB and MG-M. Wrote the paper: FB and MG-M.

## Conflict of Interest Statement

The authors declare that the research was conducted in the absence of any commercial or financial relationships that could be construed as a potential conflict of interest.

## Abbreviations

CD: celiac disease
GFD: gluten-free diet
HMW-GS: high molecular weight glutenin subunits
LMW-GS: low molecular weight glutenin subunits
LSD: least significant difference
NCGS: non-celiac gluten sensitivity
ppm: parts per million
RP-HPLC: reverse-phase HPLC
SE-HPLC: size exclusion HPLC
