# Supplementary material for: Characterization of Changes in Gluten Proteins in Low-Gliadin Transgenic Wheat Lines in Response to Application of Different Nitrogen Regimes
Source: Front Plant Sci. 2017 Feb 27;8:257. doi: 10.3389/fpls.2017.00257 (PMC5326781; doi:10.3389/fpls.2017.00257)
Supplement: Supplementary file 1 [file Table_1.DOCX]

**Table S1.** Total gliadins and glutenins content (total mg protein per pot) of low gliadin transgenics wheat and wild type lines for three different treatments of nitrogen from Experiment 1.

|  |  | **RP-HPLC** | | | | | | | | |
| --- | --- | --- | --- | --- | --- | --- | --- | --- | --- | --- |
|  |  | **Gliadins** | | | |  | **Glutenins** | | |  |
| **Line** | **N (mg)** | **ω** | **α** | **γ** | **Total** |  | **HMW** | **LMW** | **Total** | **Prolamins** |
| **BW208** | 120 | 37.6b | 88.6b | 49.1b | 175.3b |  | 37.6a | 49.5b | 87.1b | 262.4b |
|  | 360 | **79.9a** | **153.0a** | **109.5a** | **342.4a** |  | 70.5ab | 89.8ab | 160.3ab | 502.8a |
|  | 1080 | **86.3a** | **165.0a** | **112.9a** | **364.2a** |  | **76.3a** | **98.9a** | **175.2a** | **539.4a** |
|  |  |  |  |  |  |  |  |  |  |  |
| **D783** | 120 | 14.6b | 28.0b | 11.0a | 53.6b |  | 55.1b | 59.2b | 114.3b | 167.9b |
|  | 360 | 21.5ab | 42.8ab | 14.9a | 79.2ab |  | 97.0ab | 97.5ab | 194.5ab | 273.7ab |
|  | 1080 | **25.2a** | **51.4a** | 17.6a | **94.2a** |  | **116.7a** | **124.1a** | **240.8a** | **335.0a** |
|  |  |  |  |  |  |  |  |  |  |  |
| **D793** | 120 | 12.6b | 11.5b | 1.0b | 25.1b |  | 39.8b | 23.3b | 63.1b | 88.2b |
|  | 360 | 20.3b | 14.5b | 2.5b | 37.3ab |  | 76.6b | 38.3ab | 114.9b | 152.2ab |
|  | 1080 | **39.4a** | **43.7a** | **13.0a** | **96.0a** |  | **130.8a** | **73.8a** | **204.6a** | **300.6a** |
|  |  |  |  |  |  |  |  |  |  |  |
| **D577** | 120 | 22.4b | 39.7b | 4.8c | 66.9b |  | 31.3b | 39.8b | 71.1b | 138.0b |
|  | 360 | **87.3a** | **163.2a** | **18.9b** | **269.3a** |  | **74.5a** | **83.2a** | **157.7a** | **427.0a** |
|  | 1080 | **109.8a** | **212.3a** | **30.7a** | **352.8a** |  | **100.1a** | **125.0a** | **225.1a** | **577.9a** |
|  |  |  |  |  |  |  |  |  |  |  |
| **C655** | 120 | 28.6b | 52.8b | 6.6b | 88.0b |  | 36.1b | 52.2b | 88.3b | 176.3b |
|  | 360 | **91.3a** | **179.2a** | **22.7a** | **293.2a** |  | **79.7a** | **103.1a** | **182.8a** | **475.8a** |
|  | 1080 | **111.1a** | **226.0a** | **25.2a** | **362.4a** |  | **102.9a** | **134.2a** | **237.1a** | **599.5a** |

Gliadins and glutenins were determined by RP-HPLC. HMW, high molecular weight; LMW, low molecular weight.

Means with the same letter for each line and protein fraction are not significantly different as determined by LSD multiple comparisons at *p* < 0.05.
