# Supplementary material for: Characterization of Changes in Gluten Proteins in Low-Gliadin Transgenic Wheat Lines in Response to Application of Different Nitrogen Regimes
Source: Front Plant Sci. 2017 Feb 27;8:257. doi: 10.3389/fpls.2017.00257 (PMC5326781; doi:10.3389/fpls.2017.00257)
Supplement: Supplementary file 2 [file Table_2.DOCX]

**Table S2.** Storage proteins content related to N treatments from Experiment 2.

|  | **N treatment (mg)** | | | |
| --- | --- | --- | --- | --- |
|  | **µg/mg flour** | | **Total mg protein per pot** | |
| **Protein fraction** | **120** | **1080** | **120** | **1080** |
| **ω-gliadins** | 8.3b | 13.4a | 42.5b | 70.5a |
| **α-gliadins** | 8.6b | 18.0a | 45.5b | 90.3a |
| **γ-gliadins** | 2.9b | 9.0a | 16.4b | 42.4a |
| **Total gliadins** | 19.8b | 40.0a | 104.4b | 203.2a |
| **HMW** | 15.4b | 26.1a | 76.3b | 145.2a |
| **LMW** | 13.9b | 23.1a | 72.1b | 125.1a |
| **Total glutenins** | 29.3b | 49.3a | 148.4b | 270.3a |
| **Total prolamins** | 49.2b | 89.7a | 252.8b | 473.4a |
| **Gli/Glu ratio** | 0.77b | 0.84a |  |  |

Gliadins and glutenins were determined by RP-HPLC. 120, 360 and 1080 are the N treatments expressed in mg of N. HMW, high molecular weight; LMW, low molecular weight.

Samples with same letter within each protein fraction are not significantly different at *p* < 0.05 by the LSD multiple comparison of means.
