# Supplementary figures and images for: Characterization of Changes in Gluten Proteins in Low-Gliadin Transgenic Wheat Lines in Response to Application of Different Nitrogen Regimes
Source: Front Plant Sci. 2017 Feb 27;8:257. doi: 10.3389/fpls.2017.00257 (PMC5326781; doi:10.3389/fpls.2017.00257)

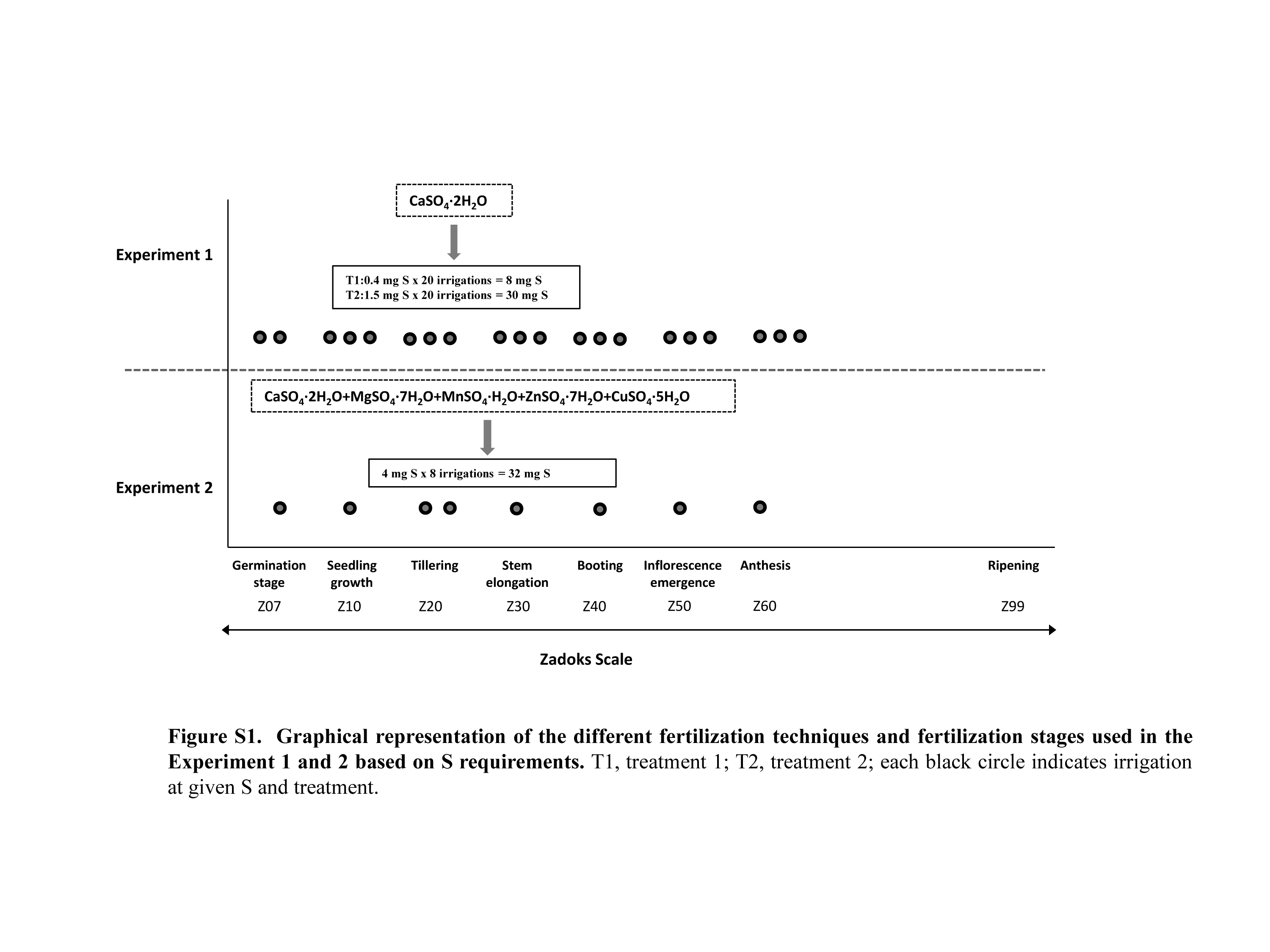

Supplement: Supplementary file 3 [file Image_1.TIF]

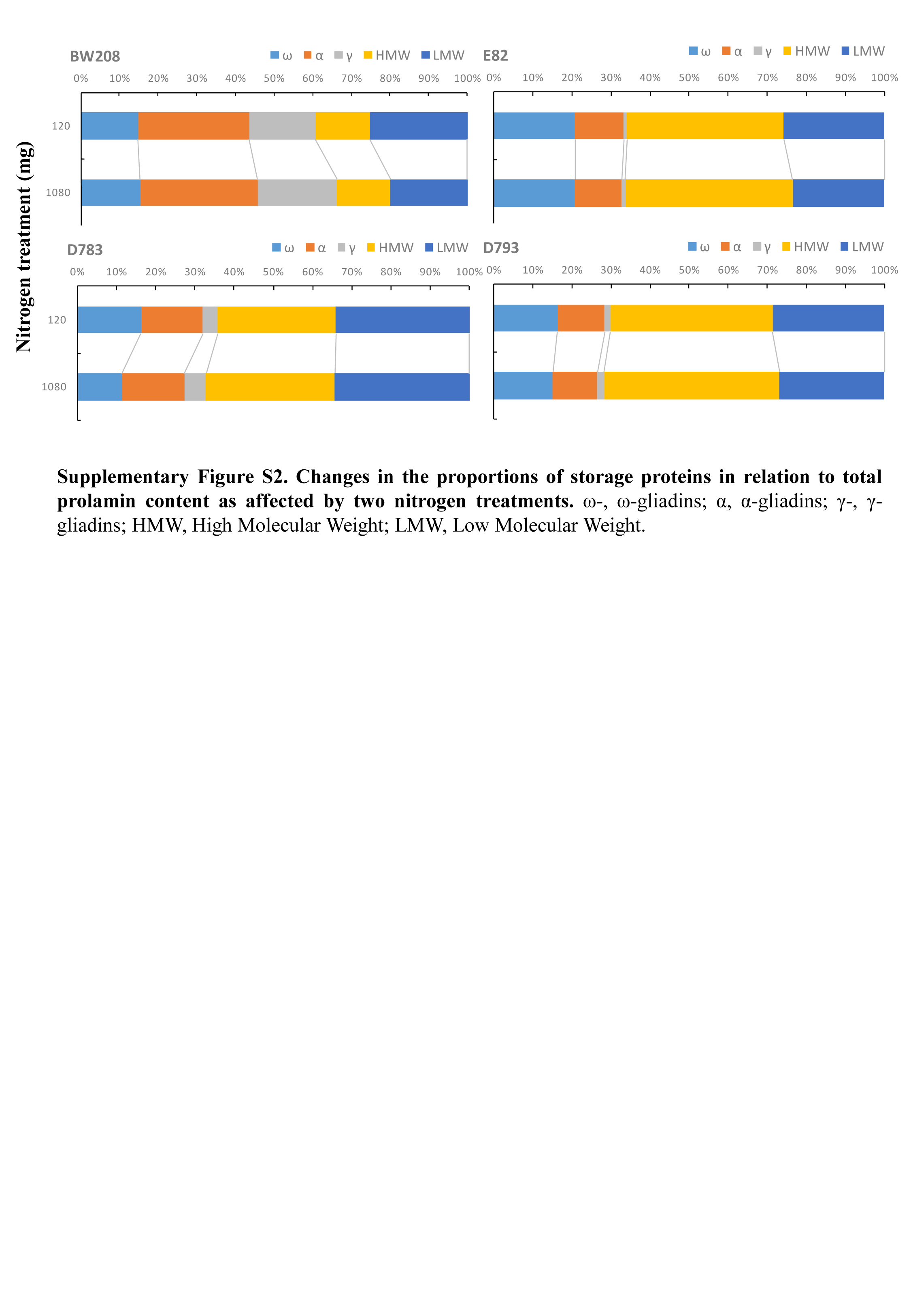

Supplement: Supplementary file 4 [file Image_2.TIF]

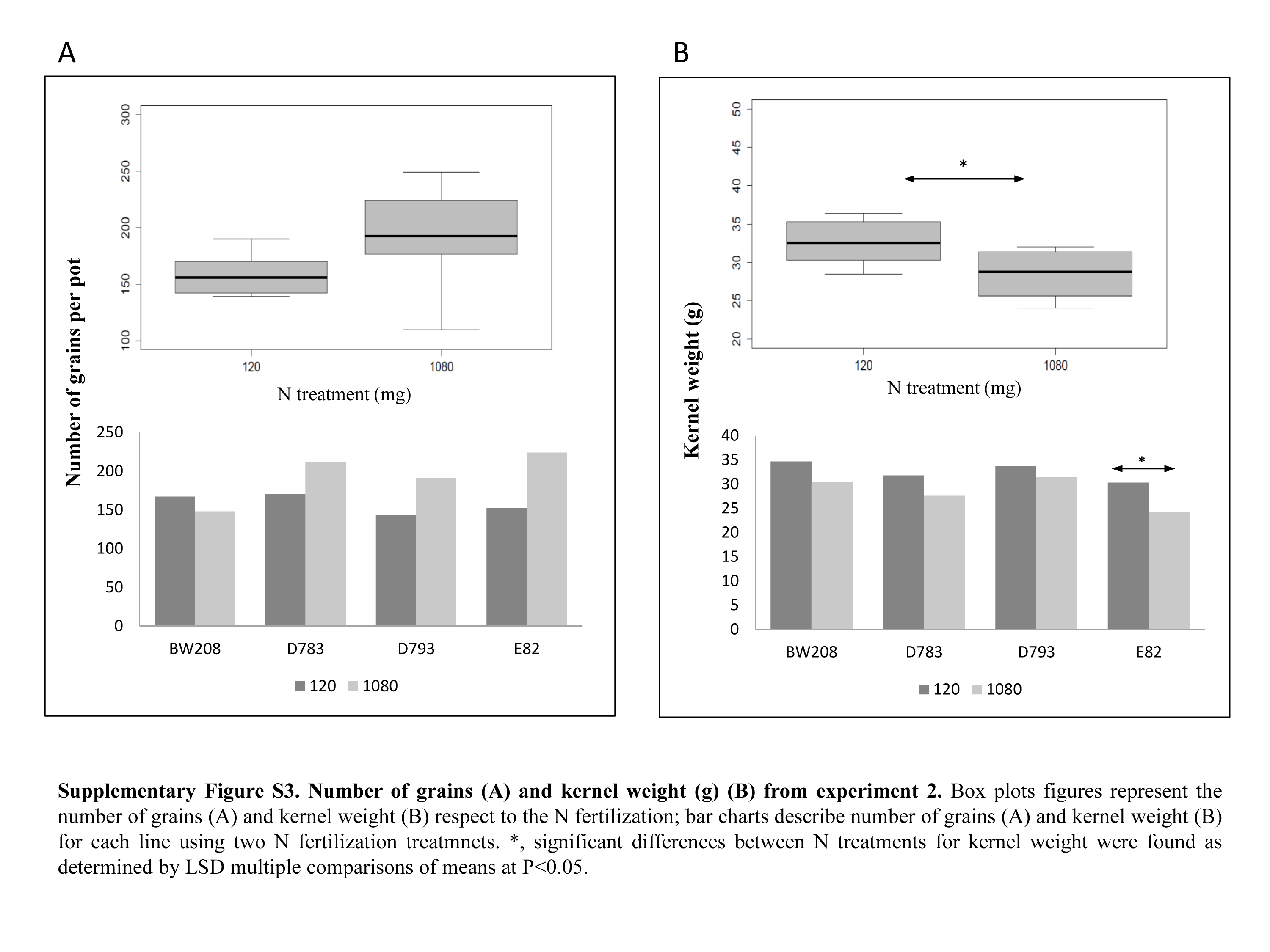

Supplement: Supplementary file 5 [file Image_3.TIF]
